# Prodigiosins from a marine sponge-associated actinomycete attenuate HCl/ethanol-induced gastric lesion via antioxidant and anti-inflammatory mechanisms

**DOI:** 10.1371/journal.pone.0216737

**Published:** 2019-06-13

**Authors:** Mohamed S. Abdelfattah, Mohammed I. Y. Elmallah, Hassan Y. Ebrahim, Rafa S. Almeer, Rasha M. A. Eltanany, Ahmed E. Abdel Moneim

**Affiliations:** 1 Natural Products Unit (NPRU), Faculty of Science, Helwan University, Ain Helwan, Cairo, Egypt; 2 Chemistry Department, Faculty of Science, Helwan University, Ain Helwan, Cairo, Egypt; 3 Faculty of Pharmacy, Helwan University, Ain Helwan, Cairo, Egypt; 4 Department of Zoology, College of Science, King Saud University, Riyadh, Saudi Arabia; 5 Zoology and Entomology Department, Faculty of Science, Helwan University, Ain Helwan, Cairo, Egypt; University of PECS Medical School, HUNGARY

## Abstract

Gastric ulcer is sores that form in the stomach mucosal layer because of erosion caused by high acid secretion and excessive use of non-steroidal anti-inflammatory drugs. Prodigiosins (PdGs) are red-pigmented secondary metabolites produced by bacteria, including actinomycetes. Butylcycloheptylprodigiosin (**1**) and undecylprodigiosin (**2**) were identified and isolated from a crude extract of the actinomycete RA2 isolated from the Red Sea Sponge *Spheciospongia mastoidea*. Chemical structure of **1** and **2** was determined by NMR and mass spectroscopy. Although their antioxidant and anti-inflammatory properties are known, their effect on gastric lesion is unknown. Therefore, this study aimed to investigate gastroprotective effects of PdGs against HCl/ethanol-induced gastric lesion in rats. Oral pretreatment with PdGs (100, 200, and 300 mg/kg) attenuated severity of HCl/ethanol-induced gastric mucosal injury, as evidenced by decreases in gastric lesion index scores, ulceration area, histopathologic abnormality, and neutrophil infiltration. These effects were comparable to those of omeprazole, a standard anti-gastric ulcer agent. HCl/ethanol-induced gastric erosions was associated with tremendous increases in lipid peroxidation, nitric oxide, and pro-inflammatory cytokines and mediators (myeloperoxidase, interleukin-1β, tumor necrosis factor-α, and cyclooxygenase-2), and with significant decreases in enzymatic and non-enzymatic antioxidant activities. However, PdGs ameliorated gastric inflammation and oxidative stress by downregulating nuclear factor kappa B and inducible nitric oxide synthase expression and upregulating heme oxygenase-1 expression. PdGs prevented gastric mucosal apoptosis by downregulating Bax and caspase-3 expression and upregulating Bcl-2 expression, thereby increasing prostaglandin E2 production. Our results suggested that PdGs exerted gastroprotective effects by decreasing the levels of inflammatory mediators, apoptotic markers, and antioxidants.

## Introduction

Gastric ulcer is a lesion that penetrates the mucosal muscularis layer characterized by inflammation, irritation, and cell loss in the gastric mucosa, can advance to gastric cancer [1]. Gastric ulcer is a global concern. Common causes of gastric ulcer include *Helicobacter pylori* infection and excessive use of non-steroidal anti-inflammatory drugs. Modern lifestyles and dietary habits, alcohol consumption, cigarette smoking, and stress are the less common causes of gastritis [2]. Inflammation and oxidative damage are commonly associated with gastric ulcer [3]. Oxidative damage is a serious problem in gastric ulcer [4]. Excessive production of reactive oxygen species (ROS) stimulates pro-inflammatory cytokine production, induces cellular protein damage, and disrupts the stomach lining, thereby causing further injury to the gastric mucosa [2]. Currently, traditional therapies for this disease include antacids and proton pump inhibitors or H2 blockers. However, the maximum recommended treatment duration for these drugs is 4 to 8 weeks and they have shown many adverse effects. Therefore, alternative therapies with antioxidants have been studied to minimize the risk of gastric ulcer and stomach cancer.

HCl/ethanol (acidified ethanol) was widely used to induce gastric mucosal injury in the animal models through enhancing ROS generation. The mechanism of ethanol-induced injury is complex and not fully understood [5]. However, HCl/ethanol causes hemorrhagic lesions, edema, epithelial exfoliation, extravasation of neutrophils, which are similar to lesions that result from alcohol abuse [6].

Actinomycetes are widely distributed in littoral zones, especially in marine animals and plants, sea sediments, and sponges. They are a phylum of Gram-positive bacteria with filamentous structure [7]. Actinomycetes are of great economical and biotechnological importance because they produce many secondary metabolites with excellent biological activities [8]. They produce 80% of known antibiotics [9], and their other metabolites exhibit diverse bioactivities, such as antifungal, antiparasitic, antioxidant, anti-inflammatory, and anticancer activities [10].

Prodigiosins (PdGs) are natural red pigments produced only in the late stages of bacterial growth. They are characterized by a common pyrrolyl pyrromethene skeleton, and produced by different types of bacteria, including actinobacteria [11]. These natural pigments have antimalarial, antibacterial, anticancer, and immunosuppressive properties [12,13]. At non-cytotoxic concentrations, PdGs inhibit murine T cell proliferation *in vitro* and *in vivo* [14]. Thus, we hypothesized that PdGs might be potential therapeutic agents for gastric lesion treatment. Thus, in the present study, we aimed to determine the gastroprotective effects of PdGs and investigate their underlying mechanisms in a rat model of HCl/ethanol-induced gastric damage.

## Materials and methods

### Collection of sponge and isolation of actinomycetes

Sponge samples were collected from the seabed at Ras Mohammed, South of Sinai, Egypt. Collection of sponge sample was achieved under the scientific collection permit 5000/2142. The permit was issued by the Nature Conservation Sector at the Egyptian Environmental Affairs Agency (EEAA). The samples were identified as *Spheciospongia mastoidea* by Prof. Rob. W. M. van Soest (University of Amsterdam, Netherlands). The fresh samples were preserved in sterile Ziploc bags containing sea water and then transported to our lab. The sponge samples were rinsed several times with sterilized sea water to remove unwanted debris, cut into small pieces, and then aseptically homogenized using sterile seawater. Next, 100 μl of diluted homogenate (10^−1^ to 10^−3^) was spread on two actinomycete-selective media, M1 and ISP2 [15]. The cultivation media used were prepared with 50% sterilized sea water supplemented with nalidixic acid (50 μg/ml) and cycloheximide (75 μg/ml) as antibacterial and antifungal agents, respectively. The plates were incubated at 28°C for 15 days until red colonies appeared. The colonies were isolated, spread on ISP2 medium, and cultured by the streaking method. The colony was identified as an actinomycete strain by morphological appearance [16] and then assigned a voucher number of RA2.

### HPLC analysis of RA2 crude extract

HPLC analysis was performed using an Agilent 1100 HPLC system equipped with a photodiode array detector. The analytical column used was an extended C18 (4.6 × 250 mm) with gradients of CH_3_CN/H_2_O at a flow rate of 0.5 ml/min. Column temperature was maintained at 25°C and the injection volume was 10 μl.

### Production of secondary metabolites from RA2

The isolated strain was cultivated on 42 Waksman agar plates at 28°C for seven days. The Waksman agar medium (100 ml) consisted of glucose (2 g), meat extract (0.5 g), peptone (0.5 g), dried yeast (0.3 g), NaCl (0.5 g), CaCO_3_ (0.3 g), agar (1.5 g), distilled water (50 ml), and sea water (50 ml). Well-grown agar plates were cut into small pieces and refluxed with MeOH (3 × 1 l). After filtration, the methanolic solution was evaporated under reduced pressure to obtain 2.6 g of red-pigmented crude extract.

### Isolation of compounds

The crude extract was fractionated using silica gel column chromatography through a stepwise gradient solvent system of increasing polarity (CH_2_Cl_2_:MeOH = 100:0, 95:5, 90:10, 85:15, 80:20, 70:30, 60:40, and 0:100) to obtain four fractions. The red bands in fraction **II** were purified by preparative thin-layer chromatography (4 plates, 20 × 20 cm, CH_2_Cl_2_/5% MeOH) to obtain compounds **1** and **2** as red solids.

### Experimental animals

Ten-week-old male albino Wistar rats weighing 170–200 g were used in the present study. All animals used in these experiments were obtained from the animal facility of the Holding Company for Biological Products and Vaccines (Cairo, Egypt). The animal were housed in wire polypropylene cages in a room under standard laboratory conditions (12-h light/dark cycle; 25±2°C). Standard rodent diet and water were available *ad libitum*. The rats were acclimated to the environment for seven days before the beginning of the experiment. All experimental procedures were approved by the Institutional Animal Ethics Committee guidelines for animal care and use at Helwan University (approval no, HU2017/Z/05), and conducted according to the European Community Directive (86/609/EEC), a national rule on animal care that is consistent with the NIH Guidelines for the Care and Use of Laboratory Animals (8^th^ edition).

### HCl/Ethanol-induced gastric lesion in rats

Thirty-six rats were randomly assigned to six groups of six rats each, and fasted for 24 h prior to oral dosing with normal saline solution (positive control), omeprazole (OMZ) 20 mg/kg (Borg Pharmaceutical Ind., Egypt; standard treatment control) [17], or PdGs at 100, 200, or 300 mg/kg. At 1 h after treatment, the rats were orally administered using an orogastric tube 5 ml/kg of a mixed solution of 0.15 M HCl (Sigma, St. Louis, MO, USA) and 60% ethanol (Sigma), as described by Son et al. [18]. Two additional control groups, the healthy control and PdGs control groups received only vehicle (normal saline solution) and 300 mg/kg PdGs, respectively, without HCl/ethanol induction. All rats were sacrificed by cervical dislocation 2 h after administration of HCl/ethanol solution under anesthesia (sodium pentobarbital, 300 mg/kg bwt, Sigma-Aldrich). The stomach was quickly removed, fixed in 4% neutral formalin solution for 1 h, opened by an incision along the greater curvature, and photographed using a Samsung WB30F camera (Samsung, Japan). Total area (mm^2^) of mucosal erosive lesion was determined using the ImageJ software version 1.50i (National Institutes of Health, USA). Percentage of lesion inhibition by PdGs was calculated by the following equation:

$$Lesion\;inhibition\;(\%)=\frac{\%L_{area}\;untreated\;lesion\;control–\%L_{area}\;PdGs-treated\;lesion\;rat}{\%L_{area}\;untreated\;lesion\;control}\times 100$$

Where %L_area_ is the percentage of ulcerated or hemorrhagic area in the total gastric mucosal area.

### Gastric acidity (pH) and gastric damage index determination

The pH of the gastric contents was determined by a digital pH meter. For gross pathology, degree of gastric mucosal damage (GDI) was estimated based on a 0–5 scoring method indicating the number and severity of gastric lesions, as previously described by Arab et al. [19]: 0 = no lesions; 1 = tiny hemorrhagic lesions; 2 = lesions < 2 mm; 3 = lesions 2–3 mm; 4 = lesions 3–4 mm; 5 = lesion > 4 mm. The score was multiplied by 2 when the width of erosion was greater than 1 mm. The mean score was calculated and expressed as GDI. GDI was identified by a blinded observer.

### Histopathological and immunohistochemical examinations

Samples of gastric tissue were collected from all rats, fixed in 10% neutral buffered formalin, processed conventionally, embedded in paraffin, sectioned to 4–5 μm thickness, and stained with hematoxylin and eosin or periodic acid-Schiff-alcian blue (PAS-alcian blue) for morphological evaluation and image analysis. Immunolocalization of NF-κB, iNOS, and TNF-α was studied. In brief, 3–4 μm-thick sections were incubated for 2 h with primary rabbit antibody against NF-κB, iNOS, or TNF-α (1:50 dilution; Santa Cruz Biotechnology, Santa Cruz, CA, USA). Primary antibodies were detected by biotin-labeled rabbit anti-mouse secondary antibody (Biotinylated Link Universal; DakoCytomation) followed by avidin/biotin-peroxidase detection solution (DakoCytomation). Gastric specimens were counterstained with hematoxylin for 1 min and mounted using Aquatex fluid (Merck KGaA, Germany). All specimens were incubated under the same conditions, at the same time, and with the same amount of antibodies to ensure that immunoreactivity would be comparable among the different experimental groups.

### Biochemical analysis of oxidative stress markers

For biochemical analyses, the glandular portion of the stomach was weighed and homogenized in an ice-cold medium containing 50 mM Tris-HCl (pH 7.4; Sigma). The homogenate was centrifuged at 1000 × *g* for 5 min at 4°C. The supernatants were used for biochemical analyses. Total protein content in the supernatants was determined according to the Lowry method [20].

Lipid peroxidation (LPO) levels in gastric homogenates were determined using the thiobarbituric acid reaction method [21] and expressed as the amount of malondialdehyde (MDA) formed. Nitrite/nitrate (nitric oxide; NO) levels were assayed by incubating the samples with Griess reagent (0.2% naphthylethylenediamine dihydrochloride and 2% sulfanilamide in 5% H_3_PO_4_) at 25°C for 10 min in the dark [22], whereas glutathione (GSH) content was determined according to the Sedlak and Lindsay method [23] using Ellman’s reagent or 5,5**ʹ**-dithio-bis-(2-nitrobenzoic acid).

### Enzymatic antioxidant status

Superoxide dismutase (SOD) activity in gastric tissue homogenates was measured as described by Nishikimi et al. [24], in which the ability of SOD to inhibit reduction of nitroblue tetrazolium to blue-colored formazan in the presence of phenazine methosulphate and NADH was measured at 560 nm. Moreover, catalase activity was estimated according to a method by Aebi [25], in which decomposition rate of H_2_O_2_ was determined at 240 nm. Furthermore, glutathione peroxidase (GPx) and glutathione reductase (GRd) activities were measured as described by Paglia and Valentine [26] and Factor et al. [27], respectively. Briefly, GPx stimulates reduction of GSH, forming oxidized glutathione (GSSG), which is then transformed back to GSH, its reduced state, by the nicotinamide adenine dinucleotide phosphate (NADPH)-dependent GRd. This reaction causes decreased absorbance at 340 nm, which is directly related to GPx activity. In contrast, estimation of GRd activity is based on the ability of GRd to oxidize NADPH, indicated by a decrease in absorbance at 340 nm monitored over 3 min.

### Myeloperoxidase activity assay

Myeloperoxidase (MPO) activity assay was performed according to the method of Bradley et al. [28]. Briefly, gastric tissue was homogenized in hexadecyltrimethyl ammonium bromide (HTMB) buffer and sonicated for 5 min. Then, the homogenate was subjected to three cycles of frozen and thawed and then centrifuged at 10000 × *g* for 30 min at 25°C. To 100 μl supernatant, 2.4 ml *O*-dianisidine hydrochloride containing 0.0005% H_2_O_2_ was added and incubated at 25°C for 10 min. The change in absorbance at 460 nm was measured spectrophotometrically and the activity of MPO was expressed as U/mg protein.

### Preparation of gastric homogenate for pro-inflammatory cytokines assay

Frozen gastric tissue was weighed and grinded with 0.1 ml ice-cold extracting buffer consisting of 0.1% IGEPAL CA-630 (a nonionic, non-denaturing detergent; Sigma) in phosphate-buffered saline and protease inhibitor cocktail (Catalog Number P8340; Sigma) at a 5:1 ratio (v/w). After grinding, the mixture was incubated for at least 10 min on ice. Next, the mixture was centrifuged for 10 min at 15,000 × *g* at 4°C. The resulting supernatant was used for PGE2, Cox-2, IL-1β and TNF-α determination. Total protein content in the supernatants was determined according to the Lowry method [20]. For the different pro-inflammatory cytokines determination, commercial ELISA kits for PGE2 (catalogue numbers: NBP1-02321), Cox-2 (catalogue numbers: NB600-971), IL-1β (catalogue numbers: NBP1-92702) and TNF-α (catalogue numbers: NBP1-92681) obtained from Novus Biologicals (Centennial, CO, USA) were used in accordance with the manufacturer’s instructions.

### Western blot analysis

We performed protein extraction and western blot analyses as described previously [29]. The utilized antibodies included goat antibody to HO-1 (AF3169, 1:500; R&D System), GAPDH (AF5718, 1:500; R&D System), goat anti-mouse IgG (sc-2039, 1:5,000; Santa Cruz Biotechnology, Santa Cruz, CA, USA). The proteins were visualized using an enhanced chemiluminescence detection kit (Bio-Rad, USA) following the manufacturer’s protocol. Images were analyzed using the Kodak Image Station 2000R (Eastman Kodak Company, Rochester, NY, USA). Protein bands intensity were normalized to GAPDH, and the data expressed in terms of percent relative to controls.

### Real-time PCR

Total RNA was extracted from gastric tissues using an RNeasy Plus Mini-kit (Qiagen, Valencia, CA, USA). First-strand cDNA was synthesized using a Script cDNA synthesis kit (Bio-Rad, CA) and amplified in 3 technical replicates using Power SYBR Green (Life Technologies, CA, USA) and an Applied Biosystems 7500 instrument. The PCR conditions were 95°C for 4 min, followed by 40 cycles of 94°C for 60 s and 55°C for 60 s, and final extension at 72°C for 10 min. After amplification, cycle number at the linear amplification threshold (Ct) values for the reference gene *Gapdh*, each sample, and relative gene expression were determined using the comparative Ct method [30]. All PCR primers used for *Hmox1*, *Bcl2*, *Bax*, *Casp3* and *Gapdh* were designed using the Primer-Blast program of NCBI and synthesized by Jena Bioscience GmbH (Jena, Germany). Primer sequences and accession numbers of the examined genes have been previously published [31] and are provided as a Supporting information (S1 Table).

### Statistical analysis

Data are expressed as the mean ± standard error of the mean (SEM). The GDI data were compared using Mann-Whitney U non-parametric test. Statistical analyses for other parameters were performed using a statistical package (SPSS version 17.0) using one-way ANOVA, and comparisons among groups were performed with the Duncan's test. Differences were considered significant at *P* < 0.05.

## Results

### Analysis of the crude extract and structure of the compounds

**Fig 1:**
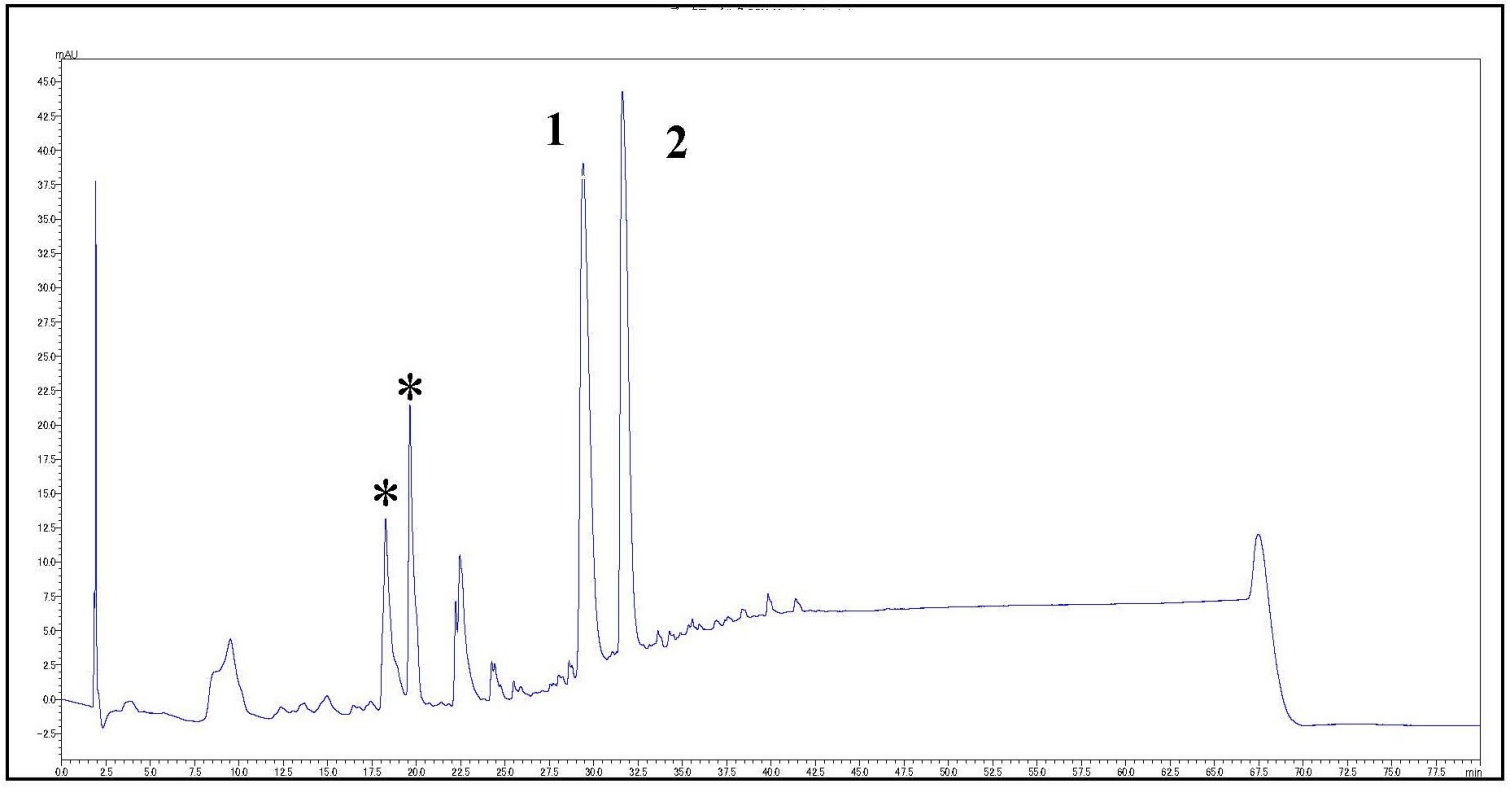
HPLC chromatogram of a crude extract of the actinomycete RA2 (peaks with asterisk were obtained from media).

**Fig 2:**
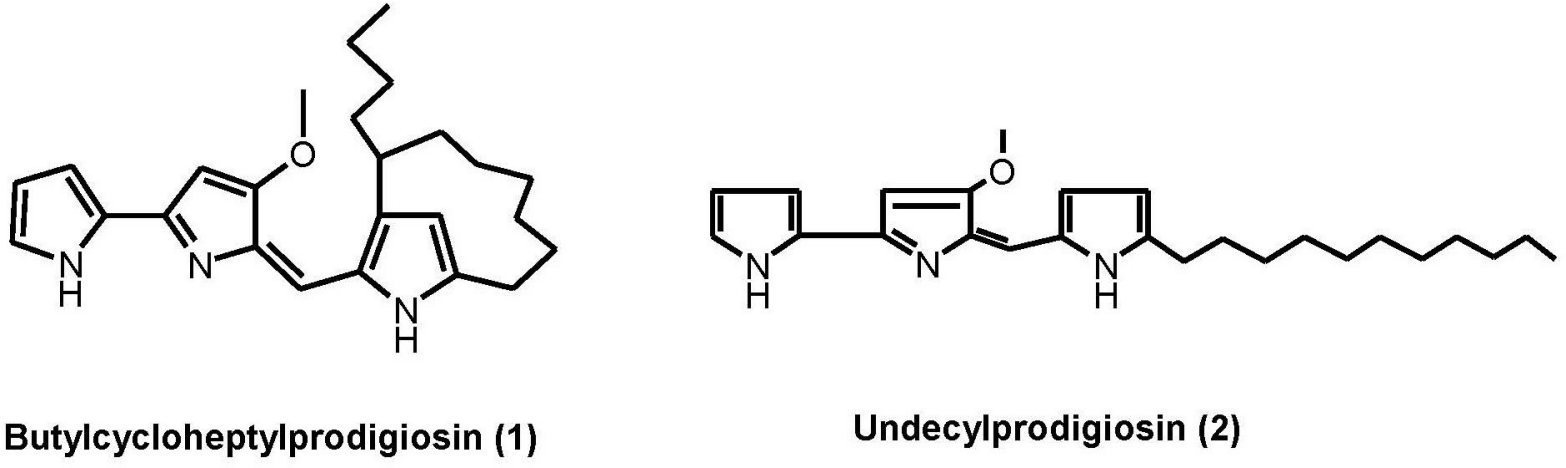
Structure of prodigiosins isolated from the actinomycete RA2. (**1**) Butylcycloheptylprodigiosin and (**2**) undecylprodigiosin.

The actinomycete RA2 was isolated from *S*. *mastoidea* collected from Ras Mohammed Protectorate, South of Sinai, Egypt. The strain was identified by morphological and microscopic examination. In our study, a microbial crude extract of the marine actinomycete RA2 showed two reddish-pink bands on TLC (DCM/5% MeOH). These bands, which are indicative of PdGs derivatives, gave yellow color after treatment with 2 N NaOH and blue color after spraying with anisaldehyde/H_2_SO_4_. HPLC analysis results of the microbial extract (Fig 1) showed the presence of two main peaks at *R*_*t*_ 30.0 and 32.5 min. Peaks number **1** and **2** showed typical absorption at a **λ**_max_ of 300 to 400 nm, indicating the presence of PdGs [32]. For upscaling, the actinomycete RA2 was cultured on several Waksman agar plates at 28°C for one week. After extraction and solvent evaporation, butylcycloheptylprodigiosin (**1**) and undecylprodigiosin (**2**) were isolated from the crude extract (Fig 2). Compound **1** was obtained as a red solid with a molecular weight of 391 Daltons, as determined by electrospray ionization mass spectrometry (ESI-MS) in the positive (*m/z* 392 [M+H]^+^; S1 Fig) and negative ion modes (*m/z* 390 [M-H]^-^; S2 Fig). The ^1^H NMR spectrum of **1** (S3 and S4 Figs) showed the presence of six olefinic or aromatic protons at δ 6.03 (1H), 6.15 (1H), 6.33 (1H), 6.78 (1H), 6.82 (1H), and 6.91(1H). Their small coupling constants (3.1 Hz) revealed the presence of heterocyclic rings. Additionally, one OCH_3_ group at δ 3.95 and several aliphatic protons were detected. By using the Dictionary of Natural Products (DNP) as a reference, we identified **1** as butylcycloheptylprodigiosin. The proton spectrum of **1** in this study is closely similar to that in the literature [32]. Compound **2** was isolated from fraction **II** as a red solid with a molecular weight of 393 Daltons, as determined by ESI-MS. Its spectrum showed *m/z* 394 [M+H]^+^ and *m/z* 833 [2M+H+2Na]^+^ in the positive ion mode (S5 Fig), as well as quasi-molecular ions at *m/z* 392 [M-H]^-^ in the negative ion mode (S6 Fig). Its ^1^H NMR spectrum (S7 and S8 Figs) in the aromatic and olefinic regions showed seven different resonance groups of 1 H, two of which were singlets at δ 6.08 and 7.10, whereas the other five were dd signals at δ 7.41, 7.20, 6.88, 6.49, and 6.32. Additionally, one methoxy group at δ 4.00 and several aliphatic protons were observed. Compound **2** was confirmed as undecylprodigiosin by comparing their NMR data with those in the literature [33].

### Effect of PdGs on gastric lesion

**Fig 3:**
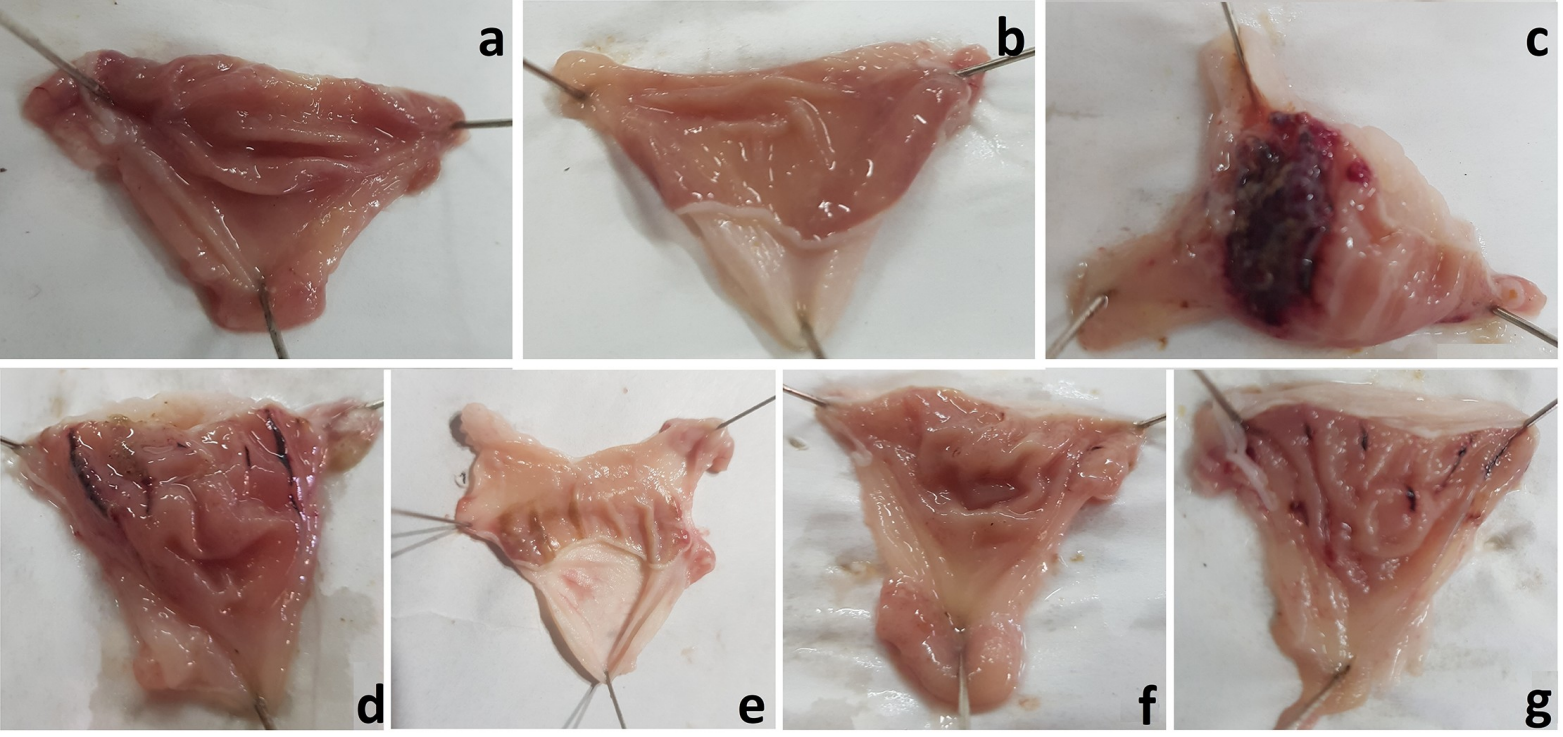
Effect of prodigiosins on gross lesions in rat stomach. (a) Stomach of control rats. (b) Stomach of the rats treated with prodigiosins (300 mg/kg) alone. (c) Stomach of the rats treated with HCl/ethanol alone. (d) Stomach of the rats pretreated with prodigiosins 100 mg/kg 1 h before HCl/ethanol treatment. (e) Stomach of the rats treated with prodigiosins 200 mg/kg 1 h before HCl/ethanol treatment. (f) Stomach of the rats treated with prodigiosins 300 mg/kg 1 h before HCl/ethanol treatment. (g) Stomach of rats treated with omeprazole (20 mg/kg) 1 h before HCl/ethanol treatment.

In the present study, HCl/ethanol-induced gastric erosions caused severe hemorrhagic lesions with large patches of mucosal necrosis and edema (Fig 3). In contrast to healthy rats, rats orally pretreated with PdGs or OMZ were obviously protected from ulceration. Gastric pH results revealed that PdGs at 100 and 200 mg/kg did not prevent the increase in pH. However, PdGs at 300 mg/kg was successfully prevented pH increase compared to HCl/ethanol-induced gastric erosion. The effect of PdGs on gastric acidity was in dose-dependent (Table 1). The GDI scores of HCl/ethanol-administered rats revealed that PdGs at the tested doses effectively prevented damage development in rats (Table 1); however, PdGs at 300 mg/kg exhibited a potent protective effect similar to that of OMZ, a standard treatment of gastric ulcer. Percentage of ulcer inhibition in 100, 200, and 300 mg/kg PdGs-treated rats was 78.6%, 90.5%, and 96.7%, respectively, whereas that in OMZ-treated rats was 92.1%.

**Table 1:** Gastric pH, gastric damage index (GDI), and % lesion inhibition (%LI) in the studied groups.

Groups	pH	GDI	%LI
**Control**	3.1±0.4	ـــــــ	ـــــــ
**PdGs (300 mg/kg)**	3.3±0.2	ـــــــ	ـــــــ
**HCl/ethanol**	2.4±0.2^a^	9.5±1.0^a^	ـــــــ
**PdGs (100 mg/kg) + HCl/ethanol**	2.7±0.3^a^^b^	1.25±0.5^a^^b^	78.6%
**PdGs (200 mg/kg) + HCl/ethanol**	2.8±0.3^a^^b^	0.9±0.42^a^^b^	90.5%
**PdGs (300 mg/kg) + HCl/ethanol**	3.0±0.4^b^	0.5±0.57^b^	96.7%
**OMZ + HCl/ethanol**	3.7±0.3^a^^b^	0.75±0.5^b^	92.1%

Values were expressed as the mean ± SD of 7 rats.

^a^*p*<0.05, significant change with respect to Control rats

^*b*^*p*<0.05, significant change with respect to HCl/ethanol-induced gastric lesion.

### Histological evaluation of gastric tissues

**Fig 4:**
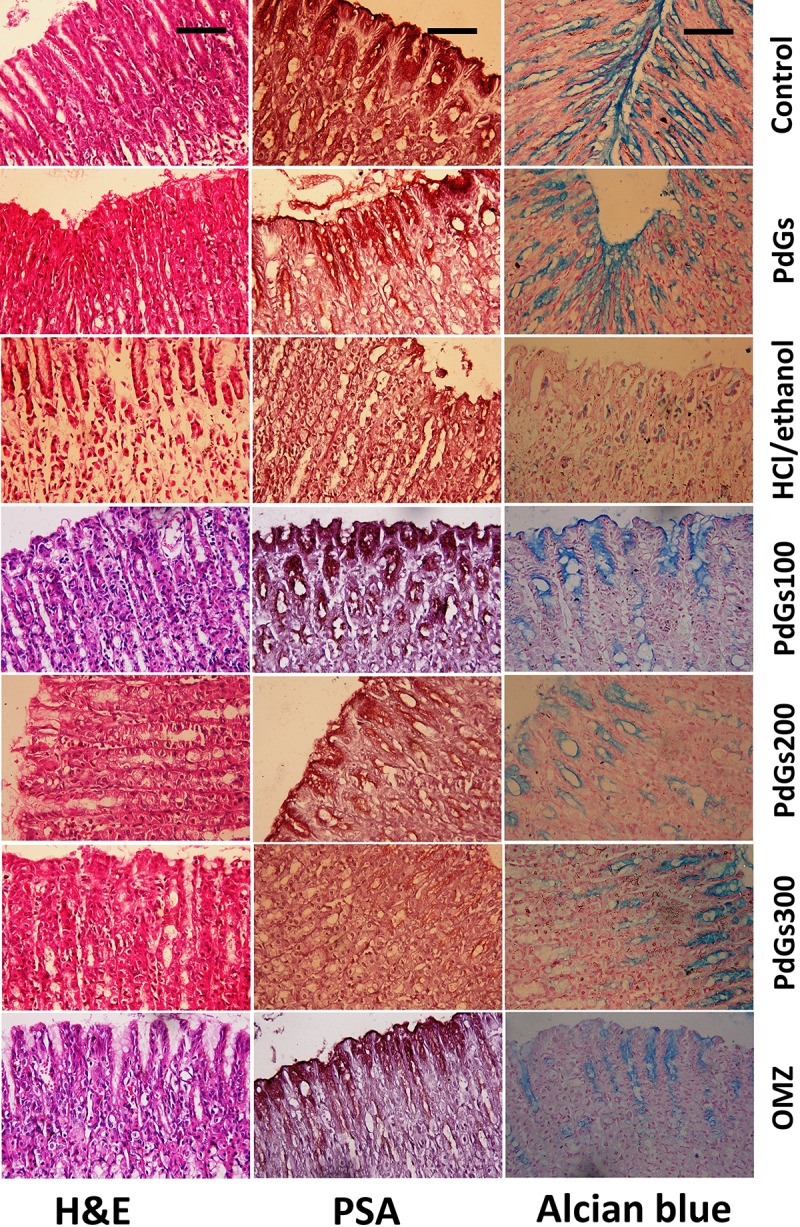
Effect of prodigiosins on histopathological alternation featurs and PAS and alcian blue reactions on HCl/ethanol-induced gastric injury in rats, where, stomach of control and prodigiosins treated alone rats showed normal structure with high mucous content. While, stomach or rats damaged by HCl/ethanol showed sever mucosal damage and hemorrhage with low mucous content. However, prodigiosins and omeprazole treatments preserved gastric layers and increased markedly mucous content (100× magnification). Scale bar = 50 μm.

Histological analyses of the healthy control and PdGs control groups revealed intact stomach wall architecture and complete mucosal layer (Fig 4). However, the rats treated with HCl/ethanol exhibited severe gastric damage, with extensively swollen submucosal layer, gastric mucosa desquamation, thinning, paleness, and erosion, as well as severely disrupted gastric glands. These lesions were associated with infiltration of inflammatory cells to all stomach layers. However, the rats pretreated with PdGs showed alleviated gastric lesions and mild gastric mucosal injury, with low desquamation of the gastric epithelial lining and low inflammatory cell infiltration. In particular, PdGs at 300 mg/kg exhibited a remarkable gastroprotective effect; the rats treated with 300 mg/kg PdGs showed normal gastric structure comparable to that observed in the rats treated with OMZ.

We further confirmed the gastroprotective effects of PdGs using PAS-alcian blue staining. The gastric mucosa of healthy control and PdGs control groups showed high intensity of PAS-alcian blue staining. On the contrary, HCl/ethanol-treated rats showed low intensity of PSA-alcian blue staining (Fig 4). However, the rats pretreated with PdGs or OMZ showed higher PAS-alcian blue staining intensity than HCl/ethanol-treated rats, indicating an increase in glycoprotein content in the stomach mucosa and attenuation of erosion in the gastric mucosal layer.

### Effect of PdGs on gastric inflammation

**Fig 5:**
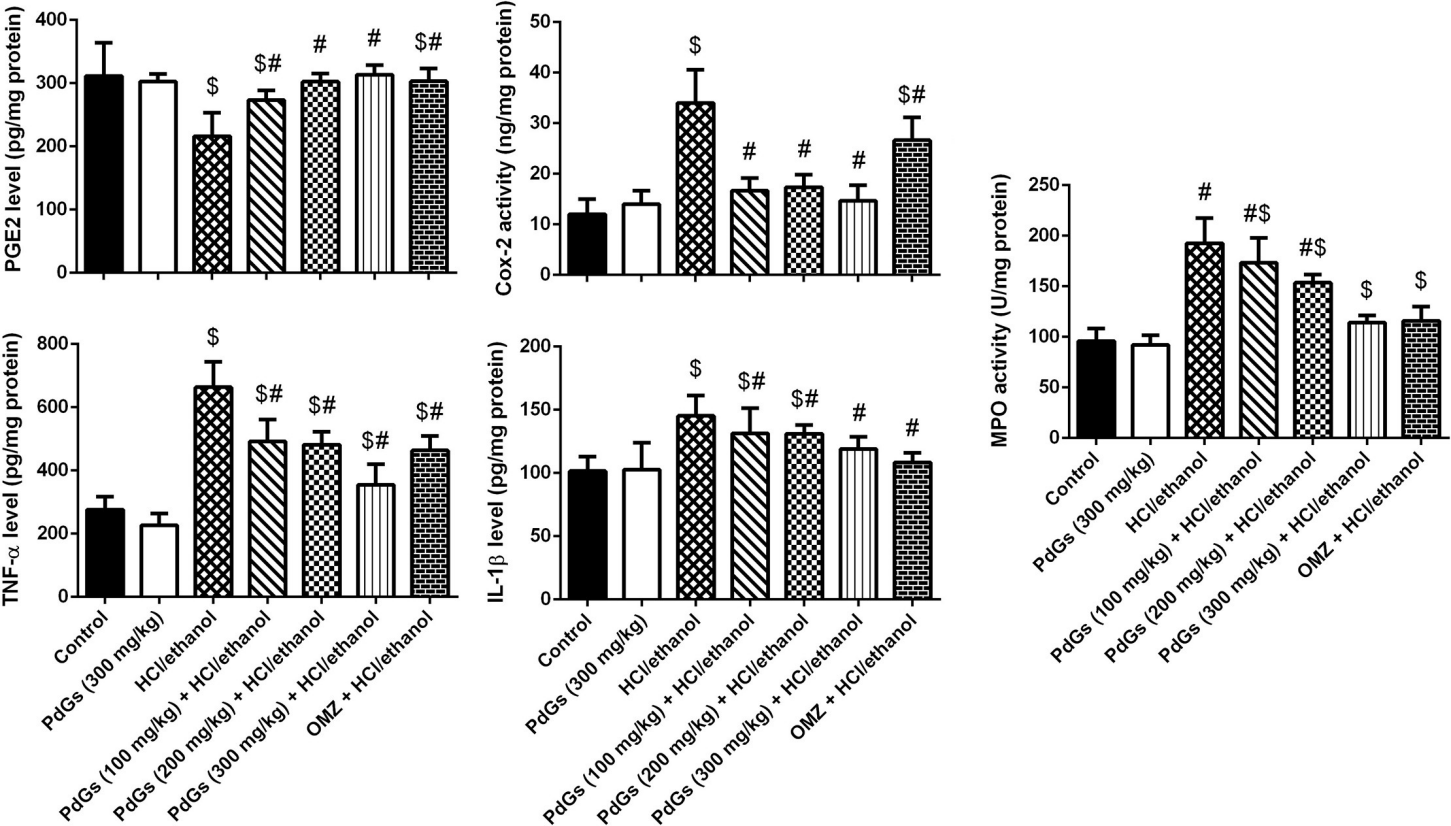
Effect of prodigiosins on the levels of the inflammatory markers PGE2, Cox-2, IL-1β, TNF-α and MPO in gastric tissue. Data are expressed as the mean ± SEM of 6 rats. ^a^*P* < 0.05, significant difference *vs*. the health rats; ^*b*^*P* < 0.05, significant difference *vs*. HCl/ethanol-induced rats, Duncan's post-hoc test.

Successful establishment of gastric lesions in rats was associated with severe inflammation indicated by significant increases in COX-2, TNF-α, and IL-1β levels, as well as a significant decrease in PGE2 level (Fig 5). However, in the rats pretreated with PdGs or OMZ, pro-inflammatory cytokine production/release was significantly lower and PGE2 production was significantly higher than in HCl/ethanol-treated rats. Additionally, MPO activity as a marker reflects neutrophils aggregation was determined in the current study. Results obtained herein revealed that MPO activity in HCl/ethanol-treated alone rats was enhanced significantly in gastric tissue. However, PdGs treatment markedly attenuated MPO activity gradually and dose dependently in the gastric tissue (Fig 5).

**Fig 6:**
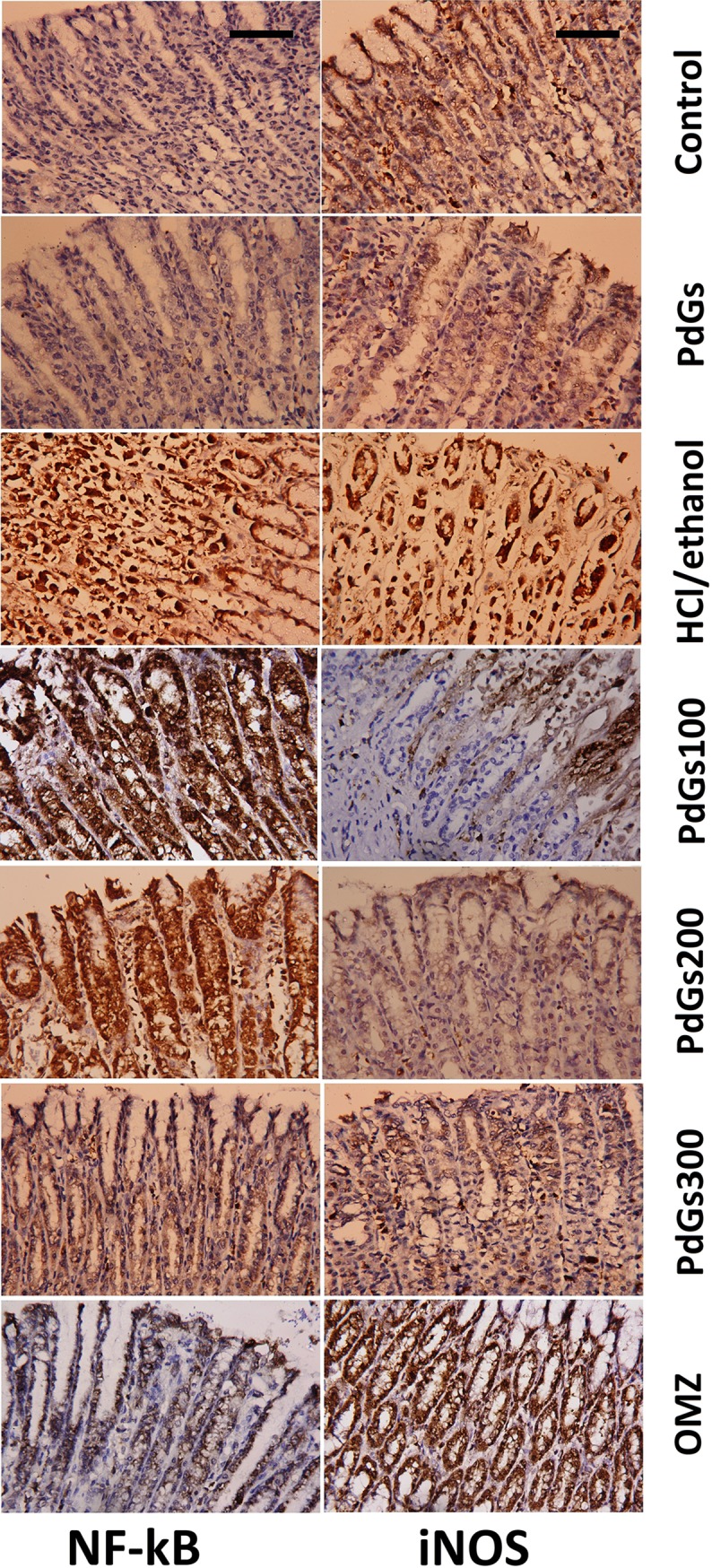
Effect of prodigiosins on the protein expression of NF-κB and iNOS in gastric tissue (100× magnification). Scale bar = 50 μm.

**Fig 7:**
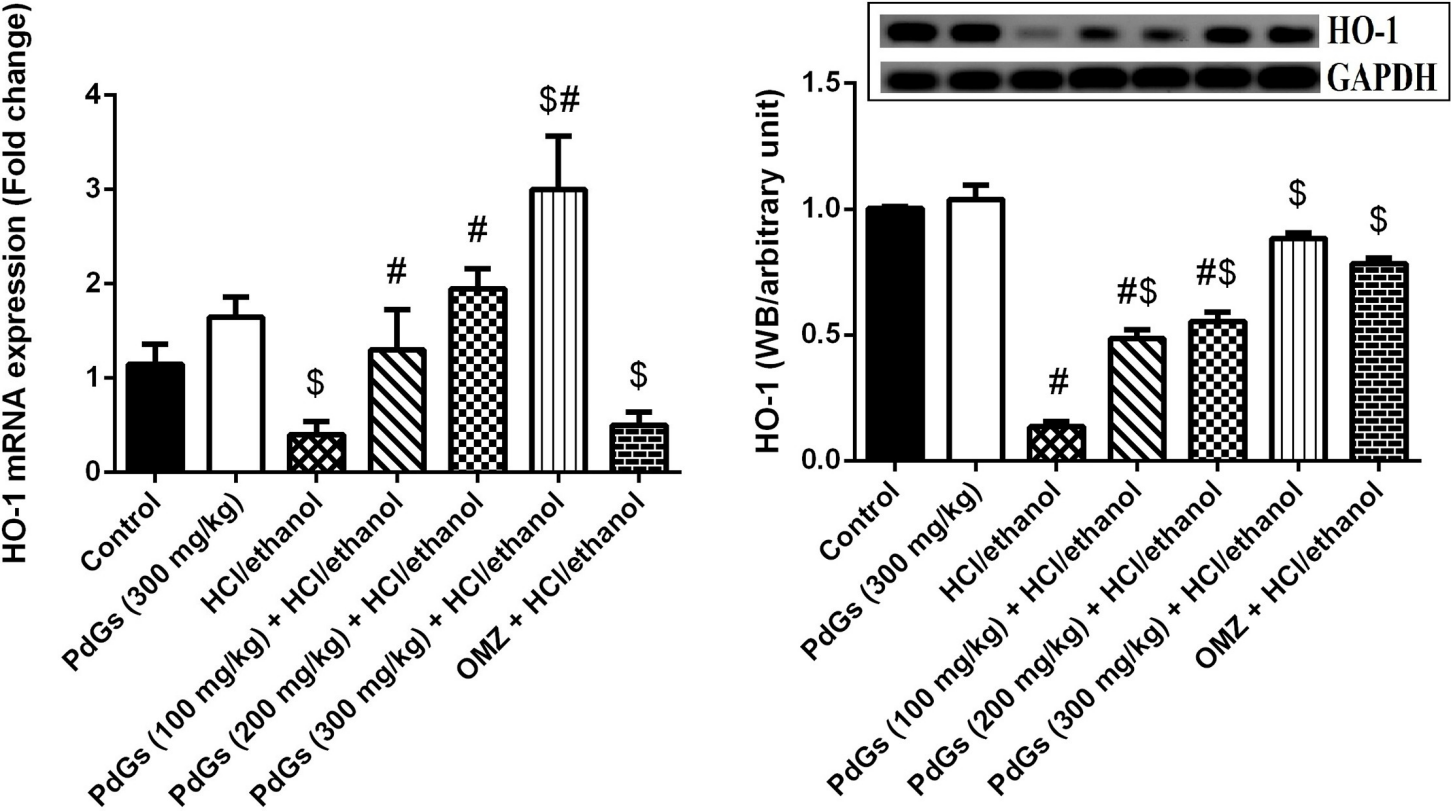
Effect of prodigiosins on the mRNA and protein expression of HO-1 in gastric tissue. Data (means ± SEM of three assays) were normalized to the levels of GAPDH and expressed as fold induction (log2 scale), relative to the level of HO-1 in the control. ^a^*P* < 0.05, significant difference *vs*. the health rats; ^*b*^*P* < 0.05, significant difference *vs*. HCl/ethanol-induced rats, Duncan's post-hoc test.

Furthermore, we showed an anti-inflammatory effect of PdGs by investigating iNOS and NF-κB expression in stomach tissues. HCl/ethanol administration caused robust inflammatory reaction, as indicated by markedly higher iNOS and NF-κB levels in the stomach tissue than those in the healthy (Fig 6). PdGs and OMZ-treated rats, however, showed markedly lower iNOS and NF-κB levels than the healthy. Furthermore, in the current study, we also examined the effect of PdGs on *Hmox1*, gene for HO-1, expression in rat gastric tissue. HCl/ethanol administration significantly suppressed HO-1 expression; however, the mRNA level of *Hmox1* was restored by pretreatment with PdGs (Fig 7). This finding was confirmed by western blotting for HO-1. The compromised of HO-1 protein by HCl/ethanol was attenuated with PdGs gradually and dose dependently.

### Antioxidant activity of PdGs

**Fig 8:**
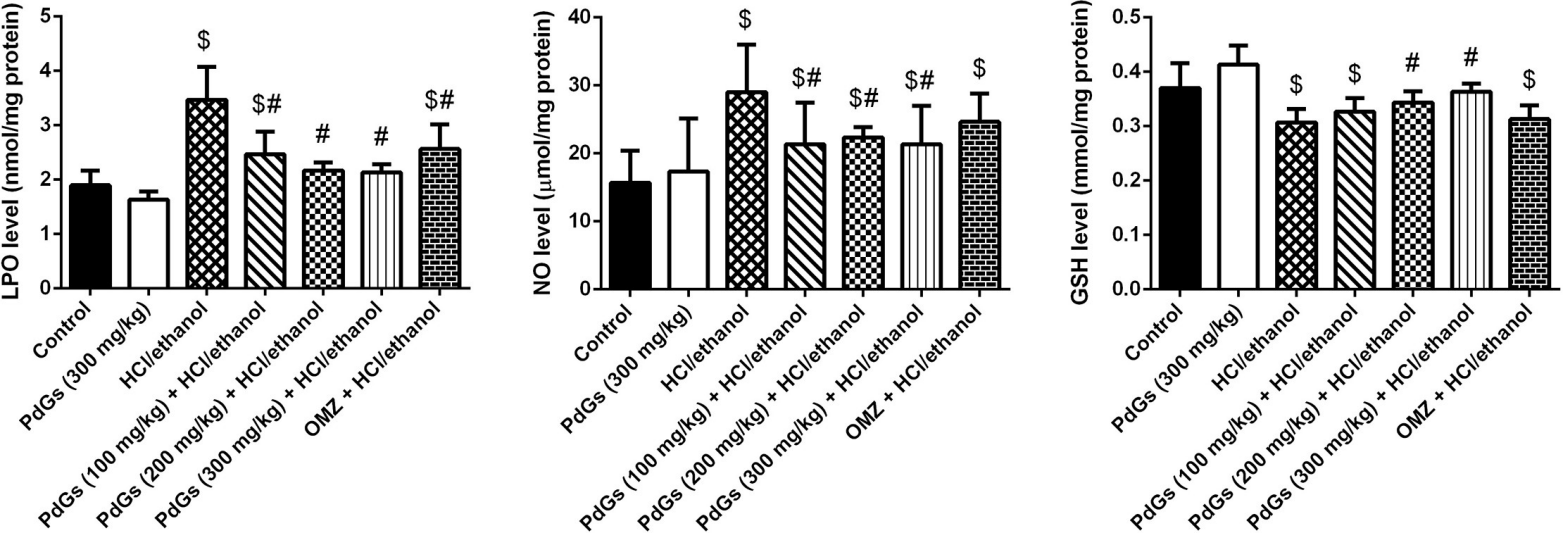
Effect of prodigiosins on LPO, NO, and GSH levels in gastric tissue. Data are expressed as the mean ± SEM of 6 rats. ^a^*P* < 0.05, significant difference *vs*. the health rats; ^*b*^*P* < 0.05, significant difference *vs*. HCl/ethanol-induced rats, Duncan's post-hoc test.

**Fig 9:**
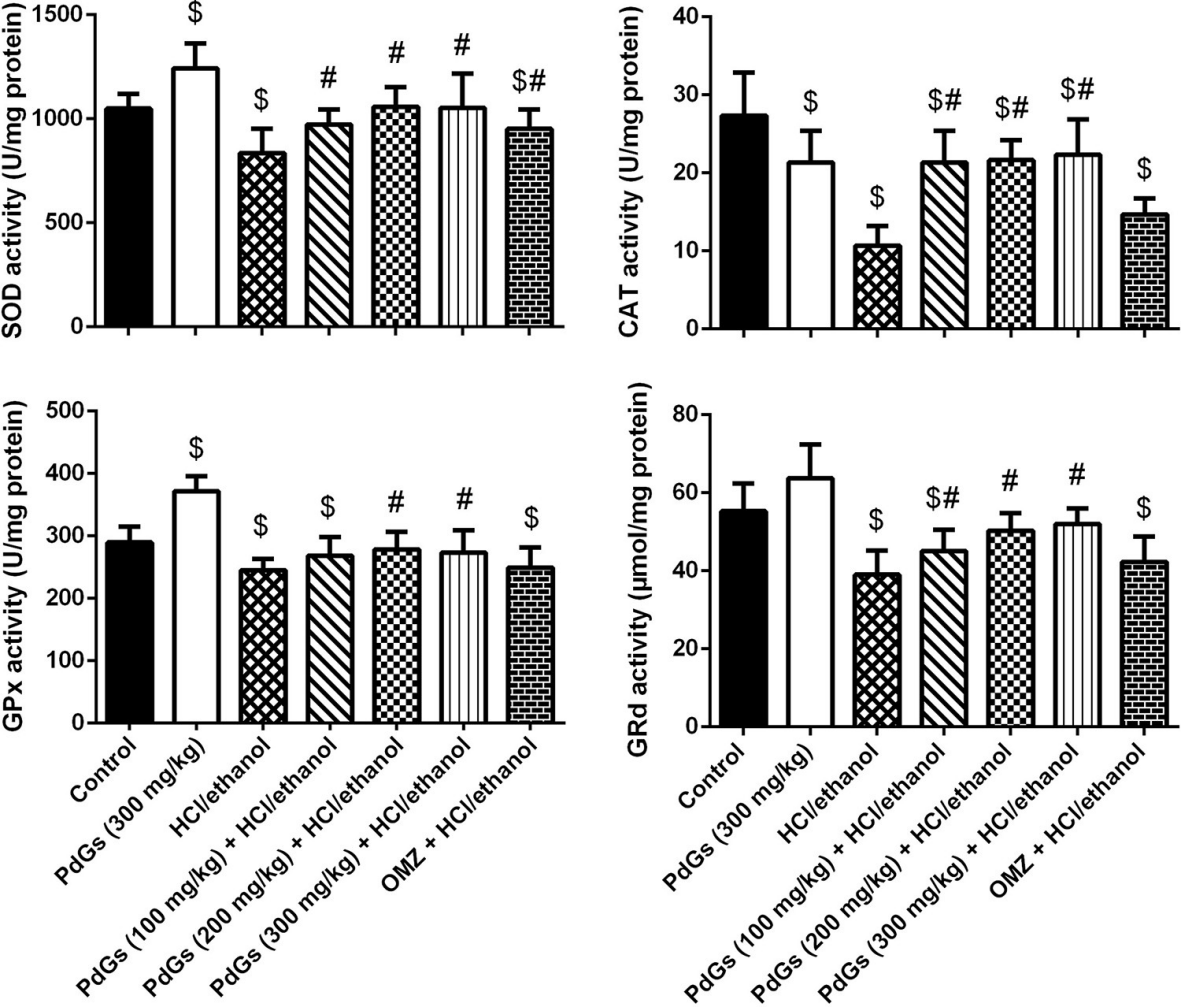
Effect of prodigiosins on SOD, CAT, GPx, and GRd activities in gastric tissue. Data are expressed as the mean ± SEM of 6 rats. ^a^*P* < 0.05, significant difference *vs*. the health rats; ^*b*^*P* < 0.05, significant difference *vs*. HCl/ethanol-induced rats, Duncan's post-hoc test.

HCl/ethanol-treated rats showed significantly higher LPO and NO levels and a significantly lower GSH content than the healthy rats (Fig 8). Furthermore, HCl/ethanol administration significantly inhibited the activities of antioxidant enzymes compared those in the healthy group. However, PdGs pretreatment prevented HCl/ethanol-induced oxidant/antioxidant imbalance by significantly increasing SOD, CAT, GPx, GRd, and GSH levels (*P* < 0.05), as well as decreasing LPO and NO levels (Figs 8 and 9).

### Antiapoptotic effect of PdGs

**Fig 10:**
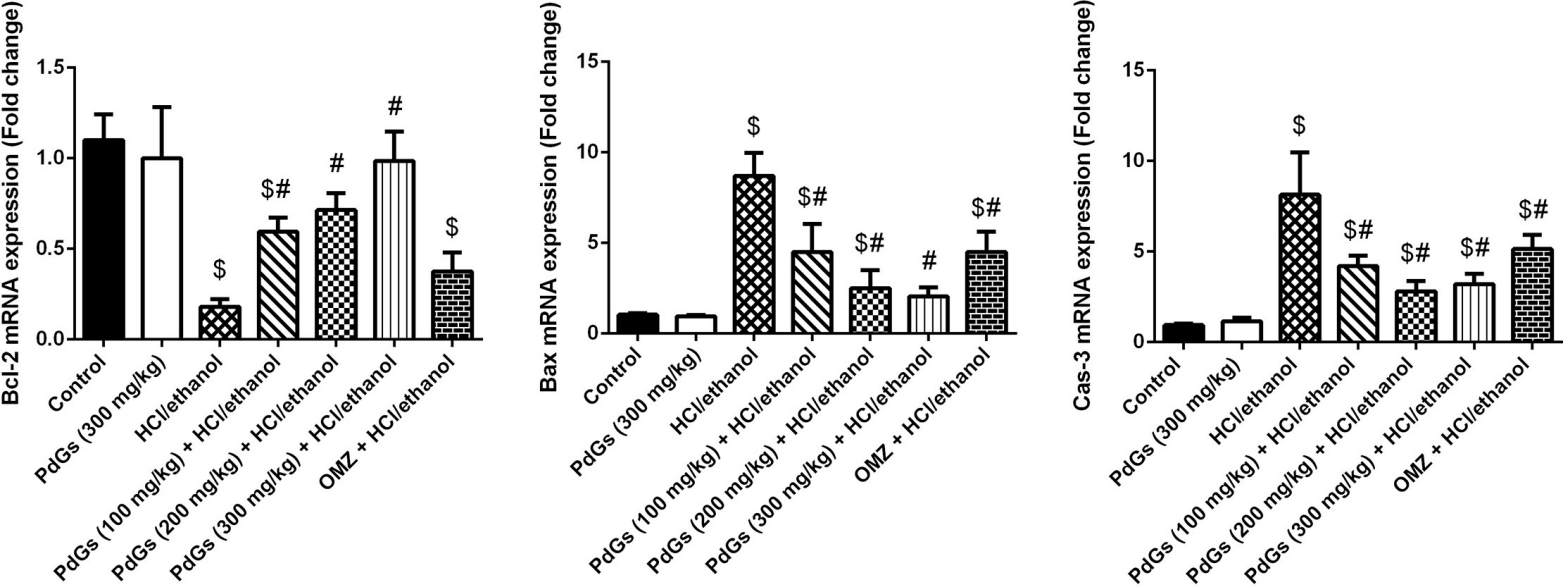
Effect of prodigiosins on the mRNA expression of *Bax*, *Bcl2*, and *Casp3* in gastric tissue. Data (means ± SEM of three assays) were normalized to the mRNA levels of *Gapdh* and expressed as fold induction (log2 scale), relative to the mRNA level of *Bax*, *Bcl2*, and *Casp3* in the health. ^a^*P* < 0.05, significant difference *vs*. the health rats; ^*b*^*P* < 0.05, significant difference *vs*. HCl/ethanol-induced rats, Duncan's post-hoc test.

qRT-PCR results revealed that in the stomach tissue of HCl/ethanol-treated rats, the mRNA expression of *Bax* and *Casp3* was significantly upregulated, whereas that of *Bcl2* was downregulated. However, pretreatment with PdGs prevented HCl/ethanol-induced apoptosis in stomach tissue (Fig 10).

## Discussion

The mucus of the alimentary canal serves as a defensive barrier against secreted HCl and other harmful substances. However, ingested noxious agents can activate neutrophil infiltration to the epithelium, which contributes to oxidative damage and gastric mucosal inflammation [1]. Ethanol stimulates H^+^-K^+^-ATP expression in the epithelial layer and increases the secretion of HCl and pepsin, the most pivotal factors in gastric epithelial damage. Besides via disruption of the mucous membrane, ethanol may also inflict gastric damage via dehydration and cytotoxic effects that lead to inflammation [19]. Another important event that occurs following HCl/ethanol administration is stimulation of neutrophil infiltration, which initiates inflammatory response, ROS production, and apoptosis [2]. In addition, a recent study reported the central role of the NF-κB signaling cascade in gastric ulcer [1]. NF-κB has many cellular roles in the regulation of innate immunity, production of cytokines, including IL-1 and TNF-α, and expression of iNOS [34]. One study reported that low expression of NF-κB causes failure of cytokine production and neutrophil recruitment in the gastric mucosa of children and adults infected with *H*. *pylori* [35]. PdGs strongly protected the gastric mucosa from HCl/ethanol-mediated gastric lesion mainly by downregulating iNOS and NF-κB expression. This was accomplished either directly via inhibition of NF-κB downstream targets, such as the pro-inflammatory IL-1β and TNF-α, or indirectly via free-radical scavenging. Interestingly, the gastroprotective effect of PdGs was comparable to that of OMZ, a reference drug currently prescribed for gastric ulcer, emphasizing the effectiveness of PdGs in attenuating HCl/ethanol-induced gastric damage associated with NF-κB expression. Supporting our findings is a study by Huh et al. [36], which reported that PdGs inhibit lipopolysaccharide-induced IκBα, a kappa B inhibitor, thereby blocking the translocation of NF-κB to the nucleus. Furthermore, the same researchers showed that PdGs reduce the protein and mRNA levels of iNOS and has a suppressive effect on the mitogen-induced proliferation of murine lymphocytes and the function of macrophage. Overexpression of iNOS leads to elevated nitric oxide production, and excess NO will react with O_2_^−^ to produce the potent oxidant peroxynitrite (ONOO^−^) [2].

Inflamed gastric epithelium and recruited leucocytes produce and release pro-inflammatory cytokines and Cox-2. In addition, IL-1β plays a pivotal role in acute inflammation response. TNF-α, a principle mediator in host response to bacterial infection, is involved in the pathogenesis of *Helicobacter spp*. infection-induced gastric ulcer. Cox-2 is a major inflammatory mediator that is activated from a quiescent state during inflammation and carcinogenesis [2]. PGE2 is a chemical mediator involved in several cellular functions, including epithelial generation, bicarbonate secretion, blood flow elevation, free radical release, and gastric motility [37]. In the current study, we showed that HCl/ethanol-induced gastric injury elevated the levels of pro-inflammatory cytokines and chemical mediators, as well as reduced PGE2 levels. In contrast, treatment with PdGs prevented these changes. PdGs selectively suppress T cell proliferation and block NF-κB activation [38]. Pradeep et al. [39] showed a promising binding efficiency of PdGs with Cox-2 using molecular docking. The inhibitory effect of PdGs on IL-1β, TNF-α, and Cox-2, as well as their stimulatory effect on PGE2, may explain their protective effects against inflammatory response induced by HCl/ethanol-mediated gastric damage and their ameliorative effect against gastric damage.

Neutrophils recruitment and aggregation in gastric mucosa is a critical process participated in gastric ulcer pathogenesis. MPO has been widely used as a biomarker of neutrophils infiltration because MPO is abundantly expressed in neutrophils. In the current study, HCl/ethanol-induced gastric injury augments MPO activity. The stimulated MPO activity is another indication of the degree of ulceration and was found to be enhanced in all the ulcer models studied. However, PdGs improved the gastric mucosal damage and enhanced healing of gastric damage involves, in part, the suppression of MPO activity. MPO utilizes the generated superoxides, leading to the formation of hypochlorous acid (HClO). HOCl is a strong oxidizer tends to mediate the generation of lipid peroxides [2]. Hence, neutrophils infiltration worsens the gastric mucosal injury and reduces with the healing process [40]. The present study reveals that PdGs has a gastroprotective effect in HCl/ethanol-induced gastric injury.

Cytokine production triggers oxidative stress. Furthermore, cytokines and oxidative stress amplify each other’s effects. During inflammation, recruited macrophages and neutrophils produce oxidants, which activate NADPH oxidase to transfer electrons to an oxygen molecule, resulting in ROS generation. ROS directly or indirectly modifies the structure of cells and macromolecules, causing oxidative damage [41]. In the present study, HCl/ethanol administration increased ROS generation, which overwhelmed cellular antioxidant defense mechanism and resulted in oxidative damage. Impairment of the antioxidant defense system was shown in the present study, as indicated by increased levels of LPO and NO and decreased activities of enzymatic and non-enzymatic antioxidants in gastric tissue. These results were supported by those of previous reports [2,42]. However, pretreatment with PdGs notably reduced LPO and NO levels and significantly enhanced the antioxidant defense system in ulcerated tissue. Recently, Arivizhivendhan et al. [43] confirmed the antioxidant potential of PdGs and showed their free radical-scavenging activity. In addition, HCl/ethanol treatment caused hemorrhagic gastric edema characterized by reddish inner layer of the gastric mucosa, mucosal erosion, and infiltration of inflammatory cells. Nevertheless, pretreatment with PdGs notably mitigated gastric lesions and inflammatory cell infiltration, confirming that PdGs exerted similar gastroprotective effects to those of the standard drug OMZ.

In the present study, HCl/ethanol-induced apoptosis of the gastric epithelium could be attributed to increased *Bax* and *Casp3* expression and decreased *Bcl2* expression. Our results were consistent with those of previous reports [19,44]. Apoptosis in the gastric epithelium was involved in HCl/ethanol-induced gastric injury and, as shown by Antonisamy et al. [45], ulcerative lesions can be ameliorated by agents that reduce cellular apoptosis by decreasing Bax and caspases expression and increasing Bcl-2 expression. Furthermore, oxidative stress and pro-inflammatory cytokines have been reported in gastric mucosal cell death [46] and attenuation of these mediators prevented apoptosis in the gastric mucosa. Additionally, enhancement of PGE2 production may attenuate gastric cell death because PGE2 has been reported to stimulate Bcl-2 [19]. Pretreatment with PdGs or OMZ significantly downregulated *Bax* and *Casp3* mRNA expression, and upregulated *Bcl2* expression, suggesting that PdGs and OMZ suppressed gastric ulceration by inhibiting apoptosis. However, PdGs exhibit proapoptotic properties in cancer cells by decreasing the expression of the surviving and Bcl-2 genes and triggering Bax and caspases-3 expression, and they also exert minimal toxicity in normal cells [47].

HO-1, an inducible enzyme that degrades heme into Fe^2+^, CO, and biliverdin, exhibits cytoprotective effects via anti-inflammatory, antioxidant, and antiapoptotic activities. Therefore, antioxidants that enhance the expression of this enzyme are considered useful to ameliorate oxidative injury [48]. In the current study, HCl/ethanol treatment suppressed HO-1 expression, which was consistent with the result of Ueda et al. [49], in which HO-1 inhibition results in increased acute gastric mucosal lesions and Gomes et al. [50] documented that HO-1/biliverdin/CO pathway played a cytoprotective role against ethanol-induced gastropathy and that these defensive effects probably result from decreased free radical generation. Magierowski et al. [51] demonstrated that HO-1 can act as the important gastroprotectants with vasoactive activities to enhance the gastric blood flow, thus conserving gastric mucosa from acetylsalicylic acid-induced gastropathy. However, pretreatment with PdGs significantly upregulated HO-1 expression, which is associated with elevated mucous production, enhanced antioxidant defense system, stabile cellular membrane, heat shock protein upregulation, and apoptosis inhibition [31]. Hence, PdGs effectively protected the gastric mucosa from HCl/ethanol-induced damage by increasing HO-1 expression.

## Conclusions

Our study proved the gastroprotective effect of PdGs, which were obtained from the marine sponge-associated actinomycete RA2, against HCl/ethanol-induced gastric injury. Our results confirmed that PdGs prevented gastric injury by inducing homeostasis of the oxidant/antioxidant and pro-/antiapoptotic signals and inhibiting NF-κB-mediated cytokine production. Moreover, the protective effect of PdGs was similar to that of the reference anti-gastric ulcer drug OMZ. These findings suggested that PdGs may be useful in the treatment of gastric damage.

## Supporting information

S1 Fig (+)-ESI-MS of butylcycloheptylprodigiosin. (DOCX)

S2 Fig (-)-ESI-MS of butylcycloheptylprodigiosin. (DOCX)

S3 Fig ^1^H NMR spectrum of butylcycloheptylprodigiosin (CDCl_3_, 600 MHz). (DOCX)

S4 Fig Expanded ^1^H NMR spectrum of butylcycloheptylprodigiosin (CDCl_3_, 600 MHz). (DOCX)

S5 Fig (+)-ESI-MS of undecylprodigiosin. (DOCX)

S6 Fig (-)-ESI-MS of undecylprodigiosin. (DOCX)

S7 Fig ^1^H NMR spectrum of undecylprodigiosin (CDCl_3_, 600 MHz). (DOCX)

S8 Fig Expanded ^1^H NMR spectrum of undecylprodigiosin (CDCl_3_, 600 MHz). (DOCX)

S1 Table Primer sequences of genes analyzed in real time PCR. (DOCX)
