# Supplementary material for: Prodigiosins from a marine sponge-associated actinomycete attenuate HCl/ethanol-induced gastric lesion via antioxidant and anti-inflammatory mechanisms
Source: PLoS One. 2019 Jun 13;14(6):e0216737. doi: 10.1371/journal.pone.0216737 (PMC6563954; doi:10.1371/journal.pone.0216737)
Supplement: S6 Fig — (DOCX) [file pone.0216737.s006.docx]

**S6 Fig:** (-)-ESI-MS of undecylprodigiosin
