# Supplementary material for: Prodigiosins from a marine sponge-associated actinomycete attenuate HCl/ethanol-induced gastric lesion via antioxidant and anti-inflammatory mechanisms
Source: PLoS One. 2019 Jun 13;14(6):e0216737. doi: 10.1371/journal.pone.0216737 (PMC6563954; doi:10.1371/journal.pone.0216737)
Supplement: S1 Table — (DOCX) [file pone.0216737.s009.docx]

**Supporting information Table S1**. Primer sequences of genes analyzed in real time PCR

| Name | Forward primer (5'---3') | Reverse primer (5'---3') |
| --- | --- | --- |
| *Gapdh* | AGTGCCAGCCTCGTCTCATA | GATGGTGATGGGTTTCCCGT |
| *Hmox1* | TTAAGCTGGTGATGGCCTCC | GTGGGGCATAGACTGGGTTC |
| *Bcl2* | ACTCTTCAGGGATGGGGTGA | TGACATCTCCCTGTTGACGC |
| *Bax* | GGGCCTTTTTGCTACAGGGT | TTCTTGGTGGATGCGTCCTG |
| *Casp3* | GAGCTTGGAACGCGAAGAAA | TAACCGGGTGCGGTAGAGTA |

The abbreviations of the genes; G*apdh*, glyceraldehyde-3-phosphate dehydrogenase; *Hmox1*: heme oxygenase 1; *Bcl2*: B-cell lymphoma 2; *Bax*, Bcl-2-like protein 4; *Casp3*, caspase-3.
